# Detection of *Mycobacterium avium ss. Paratuberculosis* in Blau Syndrome Tissues

**DOI:** 10.4061/2010/127692

**Published:** 2010-06-20

**Authors:** C. Thomas Dow, Jay L. E. Ellingson

**Affiliations:** ^1^Department of Ophthalmology and Visual Sciences, University of Wisconsin, Madison, 600 Highland Avenue, Madison, WI 53792, USA; ^2^Chippewa Valley Eye Clinic, 2715 Damon Street, Eau Claire, WI 54701, USA; ^3^Kwik Trip Inc., 2302 Kwik Trip Way, La Crosse, WI 54602, USA

## Abstract

*Background and Aim of the Work*. Blau syndrome is an inherited granulomatous inflammatory disorder with clinical findings of uveitis, arthritis, and dermatitis. Although rare, Blau syndrome shares features with the more common diseases sarcoidosis and Crohn's disease. The clinical findings of Blau syndrome are indistinguishable from juvenile sarcoidosis; the mutations of Blau syndrome are on the same gene of chromosome 16 (CARD15) that confers susceptibility to Crohn's disease. The product of this gene is part of the innate immune system. *Mycobacterium avium ss. paratuberculosis* (MAP) is the putative cause of Crohn's disease and has been implicated as a causative agent of sarcoidosis. *Methods*. Archival tissues of individuals with Blau syndrome were tested for the presence of MAP. *Results*. DNA evidence of MAP was detected in all of the tissues. *Conclusions*. This article finds that MAP is present in Blau syndrome tissue and postulates that it has a causal role. The presence of MAP in Blau syndrome—an autosomal dominant, systemic inflammatory disease—connects genetic and environmental aspects of “autoimmune” disease.

## 1. Introduction

Blau syndrome is familial juvenile systemic granulomatosis [1]. Although rare, Blau syndrome has been of interest in current medical literature because of the discovery that places its genetic defect on the same gene as one of the susceptibility genes for Crohn's disease [2, 3]. Linkage studies have placed the gene on chromosome 16; originally referred to as the NOD2 gene, it is now known as the CARD15 gene [4]. The Blau syndrome susceptibility component of the CARD15 gene is at the nucleotide binding site domain [2, 5] while the Crohn's susceptibility is at the N-terminal leucine-rich repeat domain [4, 6]. In addition to Crohn's disease and Blau syndrome, mutations of the CARD15 gene have been linked to psoriatic arthritis [7]. The CARD15 gene is part of the ancestral innate immune system that senses and eliminates bacteria [8, 9], and is part of the newly recognized, larger CATERPILLER gene family which acts as sensors to detect pathogens which regulates inflammatory and apoptotic responses [10]. Blau syndrome is unique in that it is the only systemic granulomatous disease that has a recognized Mendelian pattern of inheritance: autosomal dominant [11]. The landmark progress associating this gene with systemic granulomatous disease has prompted studies of other granulomatous disease to look for a similar defect [3, 12]. While CARD15 defects confer susceptibility Crohn's disease, Blau syndrome, and psoriatic arthritis [7] no such defects were found in patients with sarcoidosis [13], ankylosing spondylitis [14] Wegener's granulomatosis [15], systemic lupus erythematosus [16], or rheumatoid arthritis [17]. 

*Mycobacterium avium *ss*. paratuberculosis* (MAP) is an obligate intracellular organism that causes an enteric granulomatous disease in ruminant animals, Johne's disease [18, 19]. MAP is the putative cause of Crohn's disease, a similar enteric granulomatous disease of humans [20–24]. 

Traditional methods of detecting bacteria, culture, and stain, are largely ineffective in detecting MAP in humans. The bacteria are very difficult to culture and MAP is able to exist in a spheroplast (cell wall deficient) form in humans [25–27]. The advent of bacterial DNA detection with polymerase chain reaction (PCR) has greatly aided the detection of mycobacteria [28, 29]. In one series, the DNA of MAP was identified in greater than 90% of biopsy specimens from individuals with Crohn's disease [30]. Viable MAP has been recovered from the blood of patients with Crohn's disease [31]. Yet, the difficulty in culturing MAP from the blood of individuals with Crohn's is evident in a recent paper by Parrish [32] in which no samples grew MAP and, in another recent paper successful recovery of MAP was achieved in split samples [33]. In addition to Crohn's disease, MAP is also implicated in sarcoidosis [34]. Because of the association of Blau syndrome with the gene conferring susceptibility to Crohn's disease and because clinical findings of Blau syndrome are evocative of juvenile sarcoidosis, we postulated that MAP might play a role in Blau syndrome. Tissues were tested for MAP with DNA probes for IS900 and hspX. IS900 is a multicopy genetic element and is a standard probe for MAP, and hspX is very specific as it probes for a single gene specific to MAP [35].

## 2. Materials and Methods

Several clinicians who had published articles that included Blau syndrome granulomas were contacted and solicited to send representative samples for DNA probing for MAP. Three authors sent tissues: paraffin blocks of Blau skin, synovium and liver granulomas [36, 37], and unstained slides of Blau skin and renal granulomas [38]. Six different tissues from five patients representing three kindred were received and subjected to DNA probe for MAP. IS900 is the most commonly recognized DNA sequence associated with MAP and has multiple copies within the genome [18, 39]. An additional element has been found, hspX; this marker is more specific for MAP as there is only one copy within the MAP genome [35].

Tissues mounted on slides were prepared as follows: each tissue section was scraped from the glass slide into a sterile microcentrifuge tube using a sterile scalpel. Sections of the same tissue from different slides were pooled. Patient tissues embedded in paraffin were prepared as follows: blocks were effaced until complete tissue sections were obtained, then five, 5-micron sections of each paraffin-embedded tissue was cut and placed into a sterile microcentrifuge tube. Tris-EDTA (TE) buffer (100 *μ*L; pH 7.5) was then added to each tissue sample and vortexed for 1 minute. The tubes containing samples were then placed into a boiling water bath for 10 minutes and then immediately vortexed for 2 minutes. The 10-minute boiling step followed by 2 minutes of vortexing was repeated twice (a total of 3 times). Tubes were boiled for an additional 5 minutes, cooled to room temperature and then centrifuged at 10,000 rpm's for 10 minutes. DNA was extracted from 100 *μ*L of the supernatant using the Magna Pure LC DNA Isolation Kit III (Roche Diagnostics Corporation, Indianapolis, IN) according to the manufacturer's instructions. DNA was amplified according to the parameters described by Miller et al. (1999). Amplified PCR reactions were analyzed by agarose gel electrophoresis (1.5% agarose gel). All products were compared to a standard molecular weight ladder and the positive control to determine amplicon size. A sample was considered positive if amplified product was noted at 229 bp for the IS900 primer set and 211 bp with the hspX primer set. (Testing was performed by the Food Services Laboratory at the Marshfield Clinic, Marshfield, Wisconsin.) Polymerase chain reaction (PCR) [40].

## 3. Results

**Figure 1:**
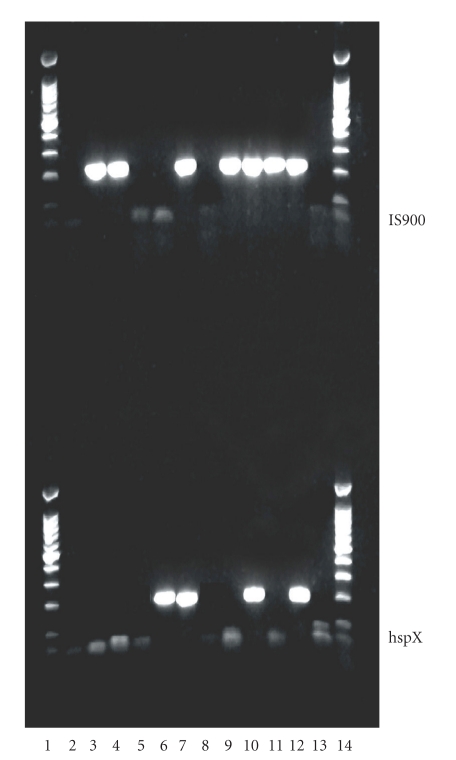
*top—IS900, bottom—hspX. *LANE 1 100 bp ladder 2–11 See Table 1. 12 Positive control MAP-ATCC 19698 13 Negative control 14 100 bp ladder.

The tested tissues represented six archival samples of Blau syndrome pathologic specimens. They were from five different patients and represented three different kindred. All tissues tested positive for IS900. Three tissues tested positive for the hspX. (Table 1 and Figure 1).

## 4. Discussion

In addition to the historic interest of MAP in Crohn's and sarcoidosis, there is recent interest in MAP as an immune trigger of several autoimmune diseases [41–43]. Increasing evidence suggests a role for MAP in autoimmune (Type 1) diabetes. It is postulated that MAP acts via molecular mimicry between genetic elements of MAP (HSP65) and the pancreatic enzyme glutamic acid decarboxylase (GAD) [44–48]. The presence of MAP DNA in Blau syndrome tissues was postulated because of the genetic connection to Crohn's disease and because of its clinical similarities to sarcoidosis—both diseases associated with MAP. Recent studies have demonstrated that sporadic cases of “juvenile sarcoidosis” are instead *de novo* CARD15 defects associated with Blau syndrome [49, 50]. This article postulates the CARD15 defect associated with Blau syndrome allows for a persistent presence of MAP. The presence of MAP in Blau syndrome-an autosomal dominant, systemic, inflammatory disease-connects genetic, and environmental (infectious) aspects of “autoimmune” disease. Larger scale studies of Blau syndrome individuals will need to be completed and include testing for MAP bacteremia to further elucidate the roll of this intriguing pathogen.

## Figures and Tables

**Table 1:** Results of DNA probing for IS900 and hpsX of *Mycobacterium avium ss. paratuberculosis* (MAP) in Blau Syndrome tissues.

Source	Rose [36]		DeChadarevian [37]		Ziegler [38]	
Patient	Mother Child		Both tissues from same patient		Mother Child	
Tissue	Liver	Synovium	Synovium	Skin	Kidney	Skin

IS900	+	+	+	+	+	+
hspX		+	+		+	
Gel Lanes	2,3,4	5	6	7	8,9,10	11
